# Nerve Blocking

**Published:** 1918-01

**Authors:** Arthur E. Smith

**Affiliations:** Special Instructor University of Tennessee, Memphis, and Loyola University. New Orleans; Dental Departments in Anesthesia and Oral Surgery


### NERVE BLOCKING

BY DR. ARTHUR E. SMITH

Special Instructor University of Tennessee, Memphis, and Loyola University, New Orleans Dental Departments in Anesthesia and Oral Surgery

The following is extracted from the lectures delivered before the Minneapolis District Dental Society in November, 1917:

The advantages of nerve blocking are numerous but the following will serve as suggestions of some of the most important:

### LONG DURATION

The duration may be changed by varying the amount of the vaso-constricting agent. The long duration is of great value to the operator for the removal of impacted third molars, reducing fractures, treating the antrum, root amputation, removal of tumors, cavity preparation, removal of pulps and many other similar operations which may come within the observation and practice of the oral surgeon or general practitioner.

2. Large or small areas may be anesthetized, depending upon the nerve branch or branches blocked.

3. Anesthesia is secured of infected or inflamed areas by blocking the nerve branch at a point some distance from the operative field and in the healthy tissue.

4. Nerve blocking injections, when skillfully made, are without pain for the reason that the needle is inserted and the solution injected in the mucous membrane and in loose connective tissue.

5. One or two insertions of the needle will block the operative field, depending, however, upon the nature of the operation and the area to be blocked.

6. Cooperation of the patient. It is well known that this is of material advantage to the operator, because he can operate with ease and complete the operation on the patient with a minimum amount of laceration and without the inspiration of blood and mucous.

### PRECAUTIONS

1. The anesthetic solution when injected should be near that of the body temperature and must be discharged slowly from the syringe and under very moderate pressure.
2. The syringe and needle should be moved back and forth slightly while discharging the solution into the tissues.
3. Better and quicker results will be obtained by having the bevel of the needle toward the nerve branch and injecting the solution toward such nerve branch or branches and not away from them.
4. The anesthetic solution must be allowed sufficient time to produce anesthesia of the nerve trunk.
5. Use iridio-platinum needles containing a high percentage of iridium and of sufficient diameter and length.
6. Use carefully selected equipment.
7. Keep the needle out of muscle tissue, ligaments and from beneath the periosteum.
8. Always make certain that you have secured complete anesthesia before operating.
9. An isotonic vehicle composed of sterile distilled water and Ringer's constituents plus a known amount of novocain and a vaso-constricting agent should be used.
10. Strict adherence to asepsis must be practiced at all times.
11. Particular attention should be paid to the bad risk patient's pulse and respiration for any pathological symptoms, both during and after injecting *a quantity* of the solution.

### CAUSES OF POST-OPERATIVE PAIN

Post-operative pain or complications are caused by one or more of the following factors:

1. By injecting a solution which is not isotonic with the blood.

2. By injecting the solution too rapidly in the tissue.

3. By injecting a solution which is not near the temperature of the body.

4. By injecting a stock solution of some form of local anesthetic which contains various ingredients or one which has undergone deterioration.

5. By using a solution which is not sterile.

6. From employing a syringe or needle which is not sterile.

7. From inserting the needle in muscle tissue, ligaments, or beneath the periosteum or from injecting the solution in the above named tissues.

8. In case post-operative pain is present following the operation it may not be due to any of the above-named factors, but may be caused from the operation itself. If the correct technique is followed, and the injecting solution consists of the proper ingredients and is properly prepared and injected, little or no pain will follow the operation.

### MANDIBULAR ANESTHESIA

### BLOCKING THE INFERIOR DENTAL AND LINGUAL NERVES

### (INTRA-ORAL METHOD)

Insert the needle through the mucous membrane, striking the inner oblique line after the external oblique line has been carefully located with the index finger. Have the distal end of the index finger resting in the *trigonum retromolare* with the dorsal surface of the finger toward the median line. Retract the mucous membrane beneath the finger in order to give ample room for the needle to pass by end of finger nail through the mucous membrane striking the inner oblique line. Have a barrel of the syringe resting over the two bicuspids on the opposite side. Be careful to keep the needle a distance of 10 millimeters above the occlusal plane of the lower teeth. This is best done by inserting the needle into the mucous membrane at the middle of the finger nail (provided your index finger is 2 centimeters in width). After the internal oblique line has been reached with the point of the needle, bring the syringe across the median line on the outside of the arch on the same side of the injection. Be careful not to allow the point of the needle to go beneath the periosteum. The approximate distance from the surface of the mucous membrane to the periosteum over the inner oblique line is 5 millimeters. When the syringe is on the outside of arch on same side of injection, insert needle posteriorly about 5 millimeters and inject one-half c.c. in order to anesthetize the lingual nerve. After this has been accomplished, bring the syringe back across the median line to its original position, or nearly so, depending upon the divergence of the two rami. Now insert the needle 10 more millimeters in order to reach the inferior dental nerve. If the syringe has been held in the proper position the point of the needle will strike the periosteum at an acute angle to the surface in the region of the inferior dental foramen, when at a depth of 20 millimeters. These measurements are approximate.

Inject 1½ to 2 c.c. for the inferior dental nerve. It is necessary for best results to work the syringe back and forth slightly at the time the solution is being discharged from the needle in order to assist the tissues in absorbing the solution and not to cause too rapid distension of the soft parts.

Amount of solution used for inferior dental and lingual nerves in the average case is 2½ c.c. The time to wait for the anesthetic to take effect will vary from ten to twenty minutes, depending a great deal upon the skill of the operator and the percentage of the solution used. The needle used for this injection is 30 millimeters long and 22 gauge. If the injection has been made properly you will find you will have complete anesthesia of the three lower molars and second biscuspids, in most cases anesthesia of the first biscuspid and in some few cases the cuspid. This depends upon how rich an anastomosis there is between the inferior dental and lingual nerves on the injected side with their fellow nerves on the opposite side.

### MENTAL ANESTHESIA

The mental foramen is located midway between the lower border and alveolar border of the lower jaw. In most individuals the foramen is located beneath the two biscuspid roots. Stand behind your patient, locate the mental foramen with the index finger by palpation or pressure. Keep this finger over the foramen during the time of injection. Depress the lip with the thumb and insert the needle into the mucous membrane at a point where the cheek blends with the gum tissue, just buccal to the second biscuspid. Direct the needle downward, inward and forward. Insert same approximately 10 millimeters. Strike the periosteum only at a depth as above described and the point of the needle should be in or near the mental foramen. Do not come in contact with the periosteum except at the foramen. Inject one or one and one-half c.c. of the solution. Massage the skin over the area of the foramen, forcing the solution through the foramen, thus anesthetizing the incisive nerve in the inferior dental canal, which is a continuation of the inferior dental nerve. You will secure anesthesia of the two biscuspids and in some cases the cuspid. This injection is sufficient for cavity preparation or pulp removal. If you extract these teeth it will be necessary to block the lingual nerve on the lingual side. The time required for anesthesia will vary from five to ten minutes if injection has been made properly. The needle used is either the one 30 millimeters in length or a 15 millimeter needle. Both needles are 23 gauge.

### INCISIVE ANESTHESIA

This injection is used for blocking the lower incisors. Insert the needle at the medium line below the roots of the two incisors at a point where the lip blends with the gum tissue. Direct the needle downward, backward and laterally to an approximate depth of 10 millimeters, striking the floor of the incisive foosa. Inject one c.c. Retract the needle without removing it from the tissue and bring the syringe to the opposite side and repeat the technique for the opposite fossa in case the nerve supply for all four incisors is desired to be blocked. The incisive fossae have numerous small forminae which transmit branches of the incisive nerve, and when the solution is injected at this point anesthesia is secured in most cases in five minutes. The needles for this injection are similar to those used for mental anesthesia. Always remember that it is necessary to block the nerve supply on the lingual side in case you desire to extract or do any kind of operative work which involves the structures on the lingual side.

### TUBEROSITY ANESTHESIA

This injection is used for blocking the posterior superior alveolar nerve which supplies the upper second and third molars and a partial supply of the first molar. This nerve also anastomoses with the dental plexus which is also formed by the middle and anterior superior alveolar nerves. The technique for this injection is as follows: Insert the needle at a point near the apex of the disto-buccal root of the upper second molar, directing it upward and backward to a point about 3 or 4 millimeters above the apex of the third molar. After the needle has been inserted into the tissue to an approximate depth of 10 millimeters, the needle should strike the periosteum over the greatest part of the curvature of the maxillary tuberosity. After you have accomplished this, follow the periosteum as closely as possible and insert the needle 10 more millimeters. The point of the needle should now be resting in the location of the entrance of the posterior superior alveolar nerves into the superior maxillary bone. (Remember that these measurements will vary slightly in different individuals.) The foraminae which give entrance to these nerves are located from 1½ to 2 centimeters above the disto-gingival margin of the upper third molar or second molar in child.) Inject 2 c.c. of the solution. You will obtain anesthesia of the upper second and third molars, and in a very small percentage of cases the first molar. The approximate time to wait for anesthesia is from 3 to 10 minutes. If you are going to remove these teeth block the anterior palatine nerve. The needle used for this injection has a slightly curved hub or shank and is 30 millimeters in length. It is 22 gauge.

### INFRA-ORBITAL ANESTHESIA

Locate the infra-orbital margin with the left index finger. Stand in front of the patient. After the infra-orbital margin has been located, bring the finger downward approximately 10 millimeters and hold same in this area during injection. The infra-orbital foramen is located 10 millimeters below the infra-orbital margin in most cases. Have your patient look directly forward and observe that your index finger is directly beneath the pupil of the patient's eye. Draw an immaginary line from the pupil of the eye to the apex of the second bicuspid and the line will pass through the infra-orbital foramen. While your index finger is resting over the foramen lift the upper lip with the thumb, thus exposing the tissue in the region of the bicuspid. Insert the needle into the mucous membrane on the buccal side of the second bicuspid at a point where the cheek blends with the gum tissue. Carry the needle upward, directing it at a point beneath the index finger. Be very careful to have the *needle parallel with the second bicuspid* (provided patient has normal occlusion), as it will give you an excellent guide in locating the infra-orbital nerve. *Do not* come in contact with the periosteum except at the time the point of the needle strikes the region in or near the foramen. Inject 2 c.c. of the solution. After a few minims of the solution have been injected you can readily detect the presence of the point of the needle and it will assist you in determining whether or not you have the needle in the right location. After the solution has been injected, gently massage the skin over the injected area, thus forcing the solution into the infra-orbital canal. This will anesthetize the anterior superior alveolar nerve, which is given off from the maxillary division of the fifth nerve in the infra-orbital canal approximately 5 millimeters posterior to the infra-orbital foramen. The success of this injection depends upon the solution reaching this nerve (anterior superior alveolar). The anterior superior alveolar nerve supplies the upper central, lateral and cuspid teeth, also anastomosing with the dental plexus located over the bicuspids and with its fellow on the opposite side. Therefore, if you require anesthesia of the central, lateral and cuspid teeth it will be necessary for you to block distal to the cuspid and distal to the central in order to block the nerve supply from the other branches. The needle used in this injection is 30 millimeters in length and 23 gauge. The time to wait for anesthesia is approximately 10 minutes. If you desire to remove these teeth, block the nerve on the lingual side.

### ANTERIOR PALATINE ANESTHESIA

This nerve enters the surface of the hard palate through the posterior palatine foramen. The posterior palatine foramen is located in most individuals midway between the linguo-gingival margin of the upper second or third molar (depending on whether the patient is an adult or child) and the median line. In other words, it is located about 15 millimeters from the linguo-gingival margin toward the median line. The anterior palatine nerve passes anteriorly along the apices of the lingual roots of the upper molars and anastomoses with the naso-palatine nerve on the lingual surface of the cuspid tooth. The technique used for this injection is as follows: Pierce the mucous membrane directly over the foramen, holding your syringe across the mouth, allowing the barrel to rest upon the bicuspids (lower) on the opposite side. Pierce the tissue at a right angle. Insert the needle approximately 10 millimeters. Insert 7 or 8 minims of the solution. The time to wait for anesthesia is from two or three minutes. You will find you have secured anesthesia, if the injection has been properly made, as far anteriorly as the distal surface of the cuspid on the lingual side. The needle used for this injection is the same as for the infra-orbital injection.

### BLOCKING THE LONG BUCCAL NERVE

The long buccal nerve is a branch of the third division of the fifth nerve. It varies in length in different individuals. I have found that at least 25 per cent. of the cases do not require blocking of this nerve for extraction of the lower second and third molars. In at least 75 per cent. of the cases this nerve sends branches to the buccal area of these two teeth. Two methods can be used for blocking this nerve. First, nerve-blocking anesthesia, which is accomplished by inserting the needle distal to the opening of Stensen's duct. Inject about 15 minims of the solution. The second method used is that of infiltration. Insert the needle into the mucous membrane near the apex of the mesial root of the lower second molar, forcing it posteriorly along the periosteum until it reaches a point distal to the third molar. Inject the solution continuously, using about 1 c.c. This is easily accomplished with the patient's mouth closed. Anesthesia is produced in from one to three minutes, the needle used being the same as for infra-orbital anesthesia.

NASO-PALATINE ANESTHESIA

The naso-palatine nerve, which is a branch of Meckel's ganglion, enters the palate through the anterior palatine foramen. It passes laterally along the ends of the roots of the central, lateral and cuspid teeth, anastomosing with anterior palatine nerve on the lingual surface of the cuspid. Insert the needle into the tissue just posterior to the most posterior papilla. Direct the needle upward and backward. Insert same from 7 to 10 millimeters and inject 7 or 8 minims of the solution. The time for anesthesia is from two or three minutes. Two needles used for this injection. One is similar to the one used for infra-orbital anesthesia, and the other is a fine, sharp one used to make initial injection.

INFILTRATION ANESTHESIA

Infiltration anesthesia is brought about by saturating the tissues in a circumscribed area with an anesthetic solution. It is called *Terminal Anesthesia* because the anesthetic acts upon the terminal branches of the sensory nerves. It is called *Regional Anesthesia* because the fluid injected affects a particular part of the sensory nerve in its course. By the use of a suitable local anesthetic the nerve elements lying in the infiltrated tissue become functionless. Infiltration anesthesia is a term used to indicate a form of local anesthesia in which the tissues are not only injected, but are distended decidedly with a fluid anesthetic indifferent in nature. If the injecting solution contains a large quantity of the anesthetic, the anesthesia is diffused for some distance beyond the point of injection, and this secondary anesthesia, or, in other words, the anesthesia which has been produced in the tissue surrounding the area infiltrated, is known as *Indirect Infiltration Anesthesia.* It will be found that the action of the anesthetic is always due to its immediate contact with the sensory nerve element, be it in the terminal nerve ending or in a nerve branch. It is self-evident that profound anesthesia can be secured much more quickly when the terminal method is employed, in comparison to the nerve blocking method; that is, injecting the solution in or near the nerve trunk, thereby requiring a longer period of time for absorption and diffusion of the anesthetic substance through the nerve trunk, which is a much larger structure than the terminal or peripheral branches. The area of anesthetized tissue depends upon the quantity of solution employed. When this method is used we depend upon the diffusion of the anesthetic solution through the minute openings of the alveolar process to anesthetize the nerves supplying the teeth.

We must consider the histology of the alveolar process in different locations of the superior and inferior maxillae. It is found that the structure of the alveolar process varies in different regions. In some regions it is very porous, while in others it is more or less dense in structure. Therefore, it is almost impossible to obtain good results from infiltration anesthesia in areas where the process is dense in structure.

The infiltration method can be employed with very good success for all teeth in the upper jaw. The dense alveolar plate which covers the cuspid, bicuspids and molars in the lower jaw inhibits the absorption of the solution, and the method is, therefore, contraindicated in most instances in this region.

### BLOCKING THE UPPER TWO BICUSPIDS

Infiltration anesthesia for these teeth in most cases in the method of choice for the dental practitioner. Insert the needle over the apex of the cuspid tooth, forcing the point down to the periosteum. Carry the needle posteriorly along the ends of the roots of the bicuspids to a point near the mesio-buccal root of the upper first molar. Be careful to keep the needle parallel with the occlusal plane of these teeth. Inject the solution continuously from the time the needle enters the tissue until it has been inserted to the proper depth. Use 2 c.c. of the solution for this injection. The needle used is the same as for the infra-orbital anesthesia. If you are going to block only one bicuspid, then use the 10 millimeter needle. Massage the gum tissue over the injected area. If you are going to remove these teeth make a lingual injection as follows: Hold the syringe across the mouth; allow the barrel to rest on the lower teeth on the opposite side. Pierce the gum tissue midway between the linguo-gingival margin and the ends of the roots of the two bicuspids. Force the needle downward forming an acute angle with the surface of the gum tissue until it strikes the periosteum then bring the needle parallel with the surface of the inner plate of the alveolar process and force the needle upward in the region of the anterior palatine nerve. The approximate depth of the needle is 15 millimeters. Inject 7 or 8 minims of the solution. The time for anesthesia is from two to five minutes. The needle used is the same as for infra-orbital injection, or the needle for infiltration.

The infiltration method is not always the method of choice. The nerve blocking method does not require numerous punctures in the mucous membrane, as does the infiltration method in case a number of teeth are to anesthetize, and enables us to anesthetize a larger area with a single injection, and the nerve trunk which is surrounded by healthy tissue is blocked at a distant point from the probably infected field of operation.

---

Dr. Smith is working out a new method of producing anesthesia of the two bicuspids and first molar (upper) by the intra-osslous method and it promises to be of greater value than the infiltration method. The new method enables the injecting of the solution without laceration or trauma to the tissue. This method can be used for any tooth or teeth.—*The Minneapolis District Dental Journal.*

